# Status of Nordic research on simulation-based learning in healthcare: an integrative review

**DOI:** 10.1186/s41077-018-0071-8

**Published:** 2018-07-04

**Authors:** Sissel Eikeland Husebø, Minna Silvennoinen, Eerika Rosqvist, Italo Masiello

**Affiliations:** 1 Faculty of Health Sciences, University of Stavanger, Stavanger, Norway; 2 Department of Surgery, Stavanger University Hospital, Stavanger, Norway; 3 Faculty of Health and Social Sciences, University of Southeast Norway, Porsgrunn, Norway; 4 School of Health and Social Studies, JAMK University of Applied Sciences, Jyväskylä, Finland; 5 Department of Teacher Education, University of Jyväskylä, Jyväskylä, Finland; 6 Department of Education and Science, The Center of Medical Expertise, Central Finland Healthcare District, Jyväskylä, Finland; 7 Department of Clinical Science and Education, Karolinska Institutet, Södersjukhuset, Stockholm, Sweden

**Keywords:** Integrative review, Nordic countries, Simulation-based learning

## Abstract

**Background:**

Based on common geography, sociopolitics, epidemiology, and healthcare services, the Nordic countries could benefit from increased collaboration and uniformity in the development of simulation-based learning (SBL). To date, only a limited overview exists on the Nordic research literature on SBL and its progress in healthcare education. Therefore, the aim of this study is to fill that gap and suggest directions for future research.

**Methods:**

An integrative review design was used. A search was conducted for relevant research published during the period spanning from 1966 to June 2016. Thirty-seven studies met the inclusion criteria. All included studies were appraised for quality and were analyzed using thematic analysis.

**Results:**

The Nordic research literature on SBL in healthcare revealed that Finland has published the greatest number of qualitative studies, and only Sweden and Norway have published randomized control trials. The studies included interprofessional or uniprofessional teams of healthcare professionals and students. An assessment of the research design revealed that most studies used a qualitative or a descriptive design. The five themes that emerged from the thematic analysis comprised technical skills, non-technical skills, user experience, educational aspects, and patient safety.

**Conclusion:**

This review has identified the research relating to the progress of SBL in the Nordic countries. Most Nordic research on SBL employs a qualitative or a descriptive design. Shortcomings in simulation research in the Nordic countries include a lack of well-designed randomized control trials or robust evidence that supports simulation as an effective educational method. In addition, there is also a shortage of studies focusing on patient safety, the primary care setting, or a combination of specialized and primary care settings. Suggested directions for future research include strengthening the design and methodology of SBL studies, incorporating a cross-country comparison of studies using simulation in the Nordic countries, and studies combining specialized and primary care settings.

**Electronic supplementary material:**

The online version of this article (10.1186/s41077-018-0071-8) contains supplementary material, which is available to authorized users.

## Background

Since the publication of the seminal book “To Err is Human” [1], which identified the need to train professionals in interprofessional teamwork as one of the many approaches to prevent medical errors, the use of SBL in healthcare has increased. SBL is used as a pedagogical method for training teamwork skills and clinical skills. SBL has since become an important technique employed to enhance quality of care and patient safety in healthcare [2–8]. Today, SBL is broadly used in several healthcare professions and in clinical practice, including graduate and postgraduate nursing education and nursing practice [4, 5, 8–11], graduate and postgraduate medical education, and medical practice [3, 12–19], health professional practice [11], and interprofessional education [7, 20–22]. In this paper, simulation is defined as “A dynamic process involving the creation of a hypothetical opportunity that incorporates an authentic representation of reality, facilitates active (participant) engagement, and integrates the complexities of practical and theoretical learning with opportunity for repetition, feedback, evaluation and reflection” ([23], p., 668).

Parallel to an increased international prevalence in the areas of both research and training, SBL has gained increasing attention in the Nordic countries: Denmark, Finland, Sweden, Iceland, and Norway [24]. These countries have common geography, sociopolitics, epidemiology, and healthcare services. A myriad of simulation activities across the Nordic countries resulted in the establishment of the “Nordic Network for Simulation-based Learning”, following the Swedish Society for Clinical Training and Medical Simulation (KlinSim.se) conference in 2016. The purpose of this Nordic network is to promote and advance simulation research in healthcare and to facilitate collaboration in research efforts and in the implementation of research-based recommendations among the Nordic countries. Scant attention has been paid in the research literature to the progress of SBL in the Nordic countries, and to date, only a limited overview exists. It has been noted in the NordForsk strategy (an organization within the Nordic Council of Ministers that finances Nordic collaboration within research) that these countries would benefit from increased collaboration and uniformity in the development of SBL [25]. In this context, the Nordic network sought to explore the status of SBL in the Nordic countries by performing an extensive, collective review of the existing Nordic simulation literature.

Therefore, the aim of the current literature review is to provide a general overview of the Nordic research literature on SBL in healthcare education and to suggest directions for future research.

The review questions addressed were as follows:

1. What is the current status of research on simulation-based learning in healthcare education?
2. Which professions have been addressed in the research on simulation-based learning?
3. Which research designs have been adopted in the research on simulation-based learning?
4. Which areas of simulation-based learning in healthcare education can be identified in the Nordic research literature?

## Methods

The integrative review was conducted following Evans’ outline ([26], p., 146). This involved a strategy comprising several stages that included a review focus, search strategy, selection criteria, critical appraisal, data collection, data synthesis, results, discussion, and analysis. This approach allowed for the inclusion of diverse methodologies in order to more fully understand the phenomenon of concern [26, 27]. To minimize bias in the review process, a review protocol with a systematic search process was developed, in accordance with Lefebvre et al. [28].

The Participants, Intervention, Comparison, and Outcomes (PICO) framework [29] was used to guide the format of the search process (see Table 1).

**Table 1:** PICO

Participants	Healthcare and education
Intervention	Integrative review
Comparison	Norway, Finland, Denmark, Sweden, Iceland
Outcomes	Simulation-based learning

### Search methods

**Fig. 1:**
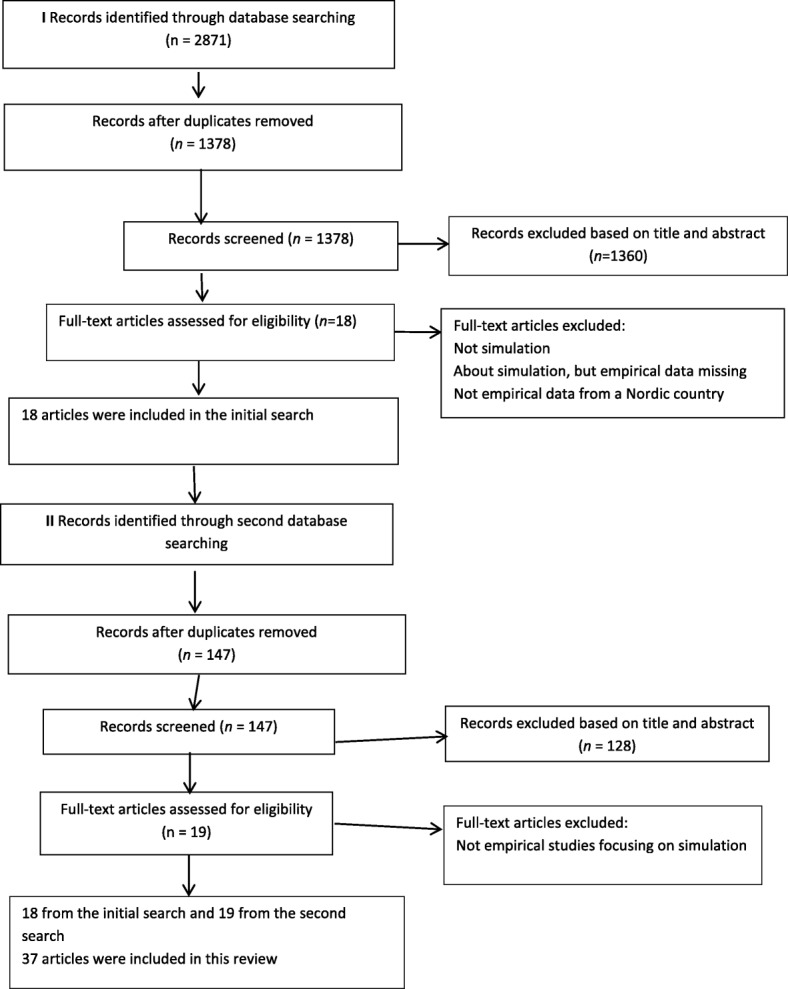
PRISMA flow chart

The first author (SEH) searched five online bibliographic databases: Academic Search Premier (ASP), CINAHL, ERIC, Medline, and SocINDEX. The following keywords were used: “Nordic”, “Norway”, “Sweden”, “Finland”, “Denmark” or “Iceland”; and “healthcare”, “nursing”, or “medicine”; and “simulation”, “teaching”, “learning”, “curriculum”, “assessment”, or “examination” (See Additional file 1). The search process was carried out during June 2016, and no limits on publication dates were set. The process of paper selection was conducted in accordance with the PRISMA flow diagram recommended by Schünemann [30] (See Fig. 1). Initially, a provisional sample of 2871 records emerged. The duplicates (1493 abstracts) were identified by SEH, resulting in 1378 records. All authors in this study separately reviewed the titles and abstracts of the articles against the following inclusion and exclusion criteria:

Included:

- Language: abstracts and papers in English, Norwegian, Danish, Swedish, Icelandic, or Finnish
- Empirical studies focusing on simulation in healthcare, nursing, or medicine
- Articles featuring empirical data material on simulation from at least one of the Nordic countries
- Peer-reviewed studies published before June 2016
- Empirical field: Studies from allied healthcare professions

Excluded:

- Abstracts from study protocols, books, and PhD theses
- Correspondence, Commentary, Letters or Debates, and Proceedings

Of the 1378 abstracts, 1360 were rejected because they did not meet the inclusion criteria, i.e., their focus was not on simulation or was about simulation but lacked empirical data, or there was no indication that the research was from one of the Nordic countries. A total of 18 full-text articles were retrieved and read by all authors, and these were included in the final selection. A second search was conducted by SEH in Svemed+, a Scandinavian database and in Medic, a Finnish database, by authors MS and ER. A sample of 147 records were found, of which 128 were rejected because they did not meet the inclusion criteria, i.e., they did not include empirical data on simulation.

### Quality appraisal

To assess the methodological quality of the studies selected, the “Mixed Method Appraisal Tool (MMAT)” [31] was used to evaluate quantitative and qualitative data [32]. The tool contains screening questions for all design methodologies, including qualitative, quantitative randomized controlled, quantitative non-randomized, quantitative descriptive, and mixed methods (Additional file 2). This tool offered a structured approach to the analysis of the research studies and assisted in the data abstraction and synthesis. To ensure that all authors possessed a similar understanding in the assessment of the studies, the tool was read and discussed before the assessment. Three of the authors (MS, ER, SEH) independently evaluated the quality of the selected studies, using the scoring system developed by Pluye et al. [31]. The format “Yes” for “present” and “No” for “not present” was used. At this stage, the authors decided to exclude eight review articles and one theoretical article because the appraisal tool was not constructed to fit these articles. The methodological quality was assessed by the “MMAT”, and the included studies scored a range from 25 to 100%. In total, 37 articles (18 from the initial search and 19 from the second search) were included in the quality appraisal.

### Data abstraction and synthesis

The 37 studies included in the data abstraction and synthesis are summarized in Table 2. Since no meta-analytic approach was appropriate due to the diverse data set and methodologies used, a thematic analysis was undertaken [33]. To facilitate the analysis, data were extracted into an evidence table. The tabulation of qualitative and quantitative findings within a single matrix supported the synthesis of both narrative and statistical data [27]. First, the 37 included articles were carefully read to obtain an overview of the entirety of the material. Second, the results of the articles were coded according to similarities and differences; the coding was then verified for accuracy and relevance by all authors [33]. Third, codes were interpreted and then grouped into categories. Finally, themes were identified. The entire synthesis was discussed among all authors in several meetings (Skype calls), until consensus was reached on the final synthesis.

**Table 2:** Data abstraction of the included studies (*n* = 37)

Authors and year	Country	Study aim(s)	Study design	Participants	Simulation modality	Key findings
Ameur et al. 2003 [63]	Sweden	To examine how contextual understanding influences performance in a virtual environment.	Mixed methods (result log, survey and in-depth interview)	*n* = 20 Medical students	Virtual reality (AccuTouch‚ Endoscopy Simulator, a bronchoscopy simulator)	The «patient group» with context manipulation perceived the simulation as more realistic than the “simulator group” did, as the latter were more focused on the psychomotor skill training.
Aspegren et al. 2006 [49]	Denmark	To determine the effect of a 13-h training in medical interviewing in pre-graduate medical education.	QUAN non-randomized (Observation) (two randomized EG and one not randomized CG)	*n* = 114 Medical students	Simulated patients (actor)	A greater proportion of the medical students in EG1 and EG2 that underwent 13 h of training achieved better medical interviewing skills than students who did not undergo such training (CG3).
Bjørshol et al. 2011 [42]	Norway	To evaluate whether socioemotional stress affects the quality of CPR during advanced life support in a simulated manikin model.	QUAN RCT (one EG with exposure to stress, one CG without exposure to stress)	*n* = 60 Paramedics	Manikins (Resusci Anne Simulator®- Laerdal Medical)	There were no significant differences in chest compression depth, compression rate, no-flow ratio, or ventilation rate between the two conditions. There was a significant increase in the subjective workload, frustration, and feeling of realism when the paramedics were exposed to socioemotional stress.
Bondevik et al. 2006 [57]	Norway	To examine how medical students view the use of an actor in communication and consultation education.	Mixed methods (survey including free text)	*n* = 188 Medical students	Simulated patients (actor)	Almost all students (97%) viewed the use of a simulated patient as beneficial, valid, realistic, and close to reality, but pointed out the danger of exaggeration and the potential to miss nuances.
Creutzfeldt et al. 2012 [41]	Sweden	To explore medical students’ retention of knowledge and skills as well as their proficiency gain after pre-training using a multiplayer virtual world (MVW) with avatars for CPR team training.	QUAN non-randomized (two EG and one CG)	*n* = 30 Medical students	Virtual reality (MVW technology with avatars)	EG2 displayed greater CPR-related knowledge than the CG3, EG1 scored in between. At start, EG1 and 2 adhered better to guidelines than CG3. Likewise, in EG2, no chest compression cycles were delivered at incorrect frequencies, whereas 54 (±44)% in CG3 (*p* < 0.05) and 44 (±49)% in EG1 were incorrectly paced; these differences disappeared during training.
Dahl Pedersen et al. 2006 [56]	Denmark	To evaluate interprofessional education in communication and collaboration in relation to ward rounds.	Mixed methods (pre-test and post-test measurements) (surveys)	*n* = 25 (12 nursing students and 13 medical students)	Simulated patients (actors)	The students evaluated the education very positively where the content and structure met their needs for interprofessional education regarding ward rounds.
Dieckmann et al. 2014 [72]	Denmark	To identify facilitators and barriers in a medicine label system to prevent medication errors in clinical use by health care professionals.	QUAL (interview)	*n* = 20 (10 nurses and 10 physicians)	Manikins (in a hospital room and a medication room)	The label design benefited from the standardized construction of the labels, the clear layout and font, and some warning signs. The complexity of the system and some inconsistencies (different meaning of colors) posed challenges, when considered with the actual application context, in which there was little time to get familiar with the design features.
Fuhrmann et al. 2009 [73]	Denmark	To evaluate the effect of multi-professional full-scale SBE of staff on the mortality and staff awareness of patients at risk on general wards.	QUAN non-randomized (pre-test and post-test measurements)	*n* = 1783 (1563 patients, 220 nurses and physicians)	Manikins	No significant differences were observed between the pre and post-intervention periods concerning the incidence of patients with abnormal vital signs, staff awareness of patients at risk, 30-day mortality or length of hospital stay among patients at risk.
Gabrielsen et al. 2016 [50]	Norway	To describe how nurses in a postoperative unit evaluated a SB communication course	QUAN descriptive (survey)	*n* = 40 Nurses	Simulated patients (nurse)	53% reported that the simulation improved their comprehension about how to use the model. 33% reported their communication skills improved after the course.
Hakoinen et al. 2014 [48]	Finland	To compare patient counseling performance in community pharmacies and health food stores by using a simulated customer buying a nutritional supplement.	QUAL (structured data collection sheet was used to document each visit)	*n* = 12 (6 personnel of the pharmacies and 6 personnel of the health food stores)	Simulated patients (educated person acting according to pre-designed scenario)	Compared to the pharmacies, the natural food stores did better since their customer service was faster and more convincing. Compared to natural food stores, the customer service remained more distant in pharmacies. In pharmacies, the information given to the customers was based on scientific facts while in natural food stores the wellbeing aspect was highlighted.
Haraldseid et al. 2015 [37]	Norway	To explore students’ perceptions of their learning environment in a CSL, and to increase the knowledge base for improving CSL learning conditions identifying the most important environmental factors according to the students.	QUAL (focus group interview)	*n =* 19 Nursing students	Manikins or simulated patients (peer students)	The study documented students’ experience with the physical (facilities, material equipment, learning tools, standard procedures), psychosocial (expectations, feedback, relations) and organizational (faculty resources, course structure) factors that affect the CSL learning environment.
Høyer et al. 2009 [51]	Denmark	To describe physicians’ behavior as team leaders in a simulated cardiac arrest during inter-hospital transfer.	QUAN descriptive	*n* = 72 Physicians	Manikins	Chest compressions were initiated in 71 cases, ventilation and defibrillation in 72. The median times for arrival of the driver in the patient cabin, initiation of ventilation and chest compressions, and first defibrillation were all less than 1 min. Medication was administered in 63/72 simulations, after a median time of 210 s. Adrenaline was the preferred initial drug administered (58/63). Tasks delegated were ventilations, chest compressions, defibrillation, and administration of medication.
Jacobsson et al. 2012 [53]	Sweden	To analyze how formal leaders communicate knowledge, create consensus, and position themselves in relation to others in the team.	Mixed methods (video-recordings and observation)	*n* = 96 (32 registered nurses, 32 enrolled nurses and 32 physicians)	Manikins (advanced human patient simulator)	Leaders used coercive, educational, discussing and negotiating strategies to work things through. The leaders used different repertoires to convey their knowledge to the team, in order to create a common goal of the priorities of the work. Changes in repertoires were dependent on the urgency of the situation and the interaction between team members. When using these repertoires, the leaders positioned themselves in different ways, either on an authoritarian or on a more egalitarian level.
Jansson et al. 2014 [46]	Sweden	To evaluate the effectiveness of patient simulation education in the nursing management of patients requiring mechanical ventilation.	QUAN RCT (questionnaire and observation)	*n =* 30 Critical care nurses	Manikins	After simulation, the average skill scores in the EG increased significantly in the final post-intervention observation. In the average skill scores, a linear mixed model identified significant time and group differences and time-group interactions between the study groups after the simulation. In contrast, the model did not identify any significant change over time or time-group interactions between groups in average knowledge scores.
Jäntti et al. 2009 [44]	Finland	To evaluate how much CPR is taught in lessons and in small groups in different institutions teaching different levels of emergency medicine providers, and to evaluate methods of teaching the different aspects of CPR quality in small groups.	QUAN descriptive (survey)	*n* = 42 (21 Institutes, 4 medical schools, 6 universities of applied science, 10 colleges, 1 emergency services college)	Manikins	The median for hours of theory lessons of CPR was 8 h (range: 2–28 h). The median for hours of small group training was 10 h (range: 3–40 h). The methods of teaching adequate chest compression rate were instructors’ visual estimation in 28.5% of the institutions, watch in 33.3%, metronome in 9.5% and manikins’ graphic in 28.5% of institutions. The methods of teaching adequate chest compression depth were instructors’ visual estimation in 33.3%, in manikins’ light indicators in 23.8% and manikins’ graphics in 52.3% of institutions.
Jensen et al. 2013 [54]	Denmark	To assess the potential benefits of a PCM for health care professionals involved in planning and coordination of patients with COPD and DM2, primarily focusing on the efficiency of the PCM, and secondary on satisfaction.	Mixed methods (survey and interview)	*n =* 18 (6 nurses, 6 General Practitioners and 6 hospital physicians)	Simulated patients (health informatics experts)	The results showed that health care professionals may benefit from a PCM. Furthermore, unexpected new possible benefits concerning communication and quality management emerged during the test and potential new groups of users were identified.
Jensen et al. 2015 [69]	Denmark	To describe a methodological approach for planning, preparing and conducting clinical simulations.	Mixed methods (case study and questionnaire)	*n =* 18 (6 nurses, 6 General Practitioners and 6 hospital physicians)	Simulated patients (health informatics experts)	Healthcare professionals can benefit from such a module. Unintended consequences concerning terminology and changes in the division of responsibility among healthcare professionals were also identified, and questions were raised concerning future workflow across sector borders. Furthermore, unexpected new possible benefits concerning improved communication, content of information in discharge letters and quality management emerged during the testing. In addition, new potential groups of users were identified.
Koponen & Pyörälä 2014 [62]	Finland	To explore medical students’ perceptions of 3 experiential learning methods, their attitudes to learning communication skills and their self-reported learning outcomes in 3 groups using different experiential methods: simulated patients, role-play, and theater in education.	Mixed methods (questionnaire, focus group interviews and a survey)	*n* = 132 Second-year medical students	Simulated patients	Most students (84%) in each group found these methods suitable for learning interpersonal communication competence. There were no statistically significant differences in students’ perceptions. According to the students, these three methods had five special elements in common: the doctor’s role, the patient’s role, reflective participation, emotional reactions and teacher’s actions. The students’ self-reported learning outcomes were communication skills, knowledge of doctor-patient communication, patient-centeredness, and awareness of interpersonal communication competence. A few students reported no learning outcomes. These self-reported learning outcomes were similar in the three groups. The medical students’ attitudes toward learning communication skills became more positive during the pilot course. There were no significant differences in students’ attitudes in the three groups before and after the course.
Lauri 1992 [52]	Finland	To describe the development and testing of a computer simulation program designed to assess the decision-making process in the public health nurses’ work in child health care.	QUAN descriptive (computer-based items)	*n* = 61 Public health nurses	Computer-based simulator	The results revealed some inconsistencies in the decision-making process with respect to the needs of the child and family as decisions were related more to the developmental stage of the child than to the unique needs of each family.
Lestander et al. 2016 [67]	Sweden	To explore the value of reflections after high fidelity simulation by investigating nursing students’ perceptions of their learning when a three-step Post-simulation Reflection Model was used.	QUAL (descriptive)	*n* = 16 Nursing students	Manikins (Laerdal® SimMan®)	The main theme in the first written reflections was identified as “Starting to act as a nurse”, with the following categories: feeling stressed, inadequate and inexperienced; developing an awareness of the importance of never compromising patient safety; planning the work and prioritizing; and beginning to understand and implement nursing knowledge. The main theme in the second written reflections was identified as “Maturing in the profession”, with the following categories: appreciating colleagues, good communication and thoughtfulness; gaining increased self-awareness and confidence; and beginning to understand the profession.
Mjelstad et al. 2007 [70]	Norway	To describe a pilot study of using simulation (MATADOR) as a method for training trauma team in leadership, communication and coordination.	QUAN non-randomized (survey and video-recordings)	*n* = 24 (12 emergency nurse, surgeons, anesthesia residents, anesthesia nurses, 12 medical students)	Virtual reality	MATADOR allowed fatal interventions during advanced treatment of a multi-trauma patient, something two of the student groups did. In comparison, the specialist groups managed to stabilize the patient without selecting CT before the life-threatening bleeding was clarified. The specialists were consistently faster than the students to introduce therapeutic treatment.
Mondrup et al. 2011 [40]	Denmark	To evaluate the feasibility of the applied method, and to examine differences in the resuscitation performance between the first responders and the cardiac arrest team.	QUAN descriptive	No. of participants, NS (Nurses, nurse-assistants, medical residents, medical interns, anesthesia residents, anesthesia nurses, orderlies)	Manikins (Resusci Anne Simulator- Laerdal Medical®)	Data from 13 of 16 simulations was used to evaluate the ability of generating resuscitation performance data in simulated cardiac arrest. The defibrillator arrived after median 214 s and detected initial rhythm after median 311 s. A significant difference in no flow ratio was observed between the first responders and the resuscitation team. The difference was significant even after adjusting for pulse and rhythm check and shock delivery.
Mäkitie et al. 2008 [64]	Finland	To develop computerized tomography and computer-based models of skull using rapid prototyping and manufacturing.	QUAL	*n =* 4 Medical students	Virtual reality (Computer-based virtual modeling using four different test models)	The models are suitable for learning rough anatomic structures, for example in basic level in medical teaching. When practicing microsurgery, the lack of microscopic details and rough structure decrease the usefulness of the model, so far. However, this method can provide first feeling to ear surgery, as was experienced by the residents.
Naess et al. 2011 [43]	Norway	To measure the quality of advanced CPR during simulation training, and to assess if the results are in accordance with the Norwegian guidelines of 2005.	QUAN descriptive	*n* = 64 (48 nurses and 16 physicians)	Manikins (Resusci Anne Simulator- Laerdal Medical®)	The quality of advanced CPR performed was in accordance with the Norwegian guidelines of 2005. The algorithm was followed correctly in almost every scenario, and the technical performance was good. Current results indicate no unnecessary hands-off time.
Poikela et al. 2015 [66]	Finland	To examine how two different teaching methods resulted in students’ meaningful learning in a simulated nursing experience.	QUAL (quasi-experimental) (video-recordings)	*n* = 52 Nursing students	Computer-based simulator	The students who used a computer-based simulation program were more likely to report meaningful learning themes than those who were exposed to a lecture method.
Reierson et al. 2013 [38]	Norway	To describe and discuss the key issues and challenges in the light of their importance for developing and implementing The Model of Practical Skill Performance.	QUAL (action research)	*n =* 6 Nursing teachers	Simulation modality NS	Six key issues and challenges were identified; anchoring the action research in the faculty structure, repeated dialog meetings between action research group and faculty, adapting the teacher and student roles in the simulation skills center, unequivocal understanding of the model as a theoretical and normative learning tool, curriculum consistency, and teachers’ engagement and enthusiasm.
Rosqvist & Lauritsalo 2013 [55]	Finland	To find out how nurses and physicians participating in SB trauma team training using a manikin perceived the training and its effects on their professional trauma management know-how.	Mixed Methods (questionnaire)	*n* = 169 Physicians	Manikins (Laerdal® SimMan®)	SB trauma team training was reported to have an effect on teamwork and communication. Most of the participants (96%) agreed that the simulation training was useful, irrespective of occupational group, length of working experience or number of simulation training sessions. The changes in working practices as a result of the training, reported by the participants on both the personal and team levels, ranged from single items of information and skills to overall professional development. The training was seen as providing a template that helped participants to remember relevant issues when caring for real trauma patients. It was also considered a useful induction method for new employees. Some of the participants with experience of SB trauma training had experienced the transfer of learned know-how from a simulation environment to clinical practice.
Saaranen et al. 2015 [68]	Finland	To produce information which can be utilized in developing the simulation method to promote the interpersonal communication competence of master-level students of health sciences.	QUAL (descriptive) (essays)	*n* = 47 (Master students of health sciences; nursing leadership, management, preventive nursing science, nurse teacher education)	Simulated patients (students)	Planning of teaching, carrying out different stages of the simulation exercise, participant roles, and students’ personal factors were central to learning interpersonal communication competence.
Salminen et al. 2014 [58]	Finland	To create a model for a virtual patient in primary care that facilitates medical students’ reflective practice and clinical reasoning.	QUAL (focus group interview)	*n =* 24 (10 primary care physicians and 14 medical students)	Computer-based simulator (Virtual patient)	Findings show good acceptance of the model by students. Use of the virtual patient was regarded as an intermediate learning activity and a complement to both the theoretical and the clinical part of education, filling out the gaps in clinical knowledge. It was regarded as authentic and the students appreciated the immediate feedback; the structure of the model was interactive and easy to follow. The VP case supported their self-directed learning and reflective ability.
Silvennoinen et al. 2016 [45]	Finland	To analyze data (videos and parametric data) that was saved to simulator as well as participants’ perceptions, collected using questionnaires.	QUAN descriptive (questionnaire)	*n =* 20 Physicians	Computer-based simulator (Endoscopy simulator-GI Mentor)	The results showed that the skills of the trainees had improved, especially with regard to fluency of endoscopy movement, time spent with clear view, and performance time. The trainees themselves assessed the improvement in their skills similarly, the greatest improvement being reported in coordination, handling the endoscope, and maintaining a clear view; while the least improvement was reported in procedure planning and paying attention to ergonomics during the procedure. Participants’ suggestions for improvement of the course included increasing personal feedback and theory lessons.
Tella et al. 2015 [39]	Finland	To explore and compare Finnish and British nursing students’ perceptions of learning about patient safety in academic settings to inform nursing educators about designing future education curriculum.	QUAN descriptive (questionnaire)	*n* = 195 Nursing students	Simulation modality NS	Both student groups considered patient safety education to be more valuable for their own learning than what their programs had provided. Training patient safety skills in the academic settings were the strongest predictors for differences, along with work experience in the healthcare sector. To prepare nursing students for practical work, training related to clear communication, reporting errors, systems-based approaches, interprofessional teamwork, and use of simulation in academic settings require comprehensive attention, especially in Finland.
Thesen et al. 2004 [59]	Norway	To describe a course in CPR for healthcare workers in primary care.	QUAN descriptive	*n* = 141 (32 physicians, 17 nurses, 92 medical secretaries, paramedics, assistant nurses, personal assistants)	Manikins (computer based)	54 of 61 who responded to the survey said that it was useful education in their daily work. 5 wanted this course every other year or less seldom, while the rest wanted the course once or twice a year. More than half of the participants answered that the theory, practical exercises and the level of theory was suitable.
Toivanen et al. 2012 [61]	Finland	To describe psychiatric nurses’ experiences of patient simulation as teaching and learning method in in-service training in somatic emergencies.	QUAL	*n* = 8 Psychiatric nurses	Manikins	The results indicated that patient simulation was a versatile and effective method of in-service training in somatic emergencies for psychiatric nurses. The nurses felt the teacher had an important role in promoting a positive learning atmosphere and it was important that their work profiles were taken into account in designing the simulation scenarios. The nurses thought they could utilize the education in developing their work and pointed out the need for regular training in somatic emergencies, using patient simulation to ensure their competence.
Utsi et al. 2008 [47]	Norway	To evaluate a method for interprofessional training in emergency handling, receiving and stabilization of seriously injured patients in primary care.	Mixed methods (pre-test and post-test)	*n* = 119 (27 physicians, 44 nurses, 48 paramedics, medical secretaries, assistant nurses)	Manikins	The participants reported a significant increase in their confidence in their own role and with the order of necessary actions. 91% would recommend the course to peers.
Westfelt et al. 2010 [71]	Sweden	To present health care professionals’ perception of collaboration and safety in the emergency department, and to demonstrate how such teamwork for safety can be implemented.	QUAN non-randomized	*n* = 55 (22 nurses, 13 assistant nurses, 3 anesthesia nurses, 6 surgeons, 2 urologists, 9 anesthesia residents)	Manikins (Laerdal® SimMan®)	The participants evaluated interprofessional team training with simulation as very satisfactory. Nurses and nurse assistants addressed the need for a deeper collaboration by employing an established training method in their own working environment. It is possible to train teams in a realistic environment without using real patients.
Wisborg et al. 2009 [36]	Norway	To examine the participants’ assessment of their educational outcome after training with either a simulated patient or simple resuscitation manikin.	QUAN RCT (survey and focus group)	*n* = 104 (32 physicians, 53 nurses, 19 radiographers and lab technicians)	Manikins and simulated patients (nurse or a medical student)	Participants assessed their educational outcome to be high, unrelated to the order and appearance of patient model. There were no differences in assessment of realism or feeling of embarrassment. Participants felt that the choice between the educational modalities should be determined by the simulated case, with high interaction between teams, and interaction enhanced by using a standardized patient.
Østergaard et al. 2008 [35]	Denmark	To describe a framework for the development of a team training course and to describe the development of multiprofessional team training in Denmark and its connection to patient safety.	QUAN non-randomized	No. of participants, NS Nurses, surgeons, anesthetists, radiologists, secretaries and hospital orderlies	Manikins and simulated patients	The use of the framework was illustrated by the existing multiprofessional team training in advanced cardiac life support, trauma team training, and neonatal resuscitation.

*CG* control group, *CPR* cardio-pulmonary resuscitation, *CSL* clinical skills laboratory, *EG* experimental group, *No* number, *NS* not stated, *PCM* Planning and Coordination Module, *QUAL* qualitative, *QUAN* quantitative, *RCT* randomized control trial, *SB* simulation-based, *SBE* simulation-based education

## Results

### Characteristics of the literature

**Fig. 2:**
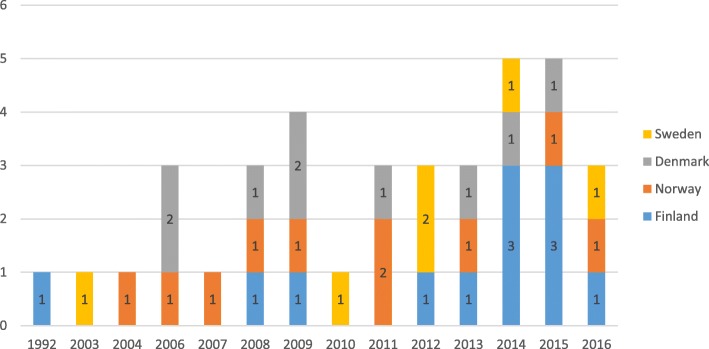
Number of papers (*n* = 37) published per year per country from 1992 to 2016

This integrative review identified 37 studies of SBL from the Nordic countries. The first Nordic simulation study included in this review was published in 1992, and the number of studies published since then generally has increased each year (Fig. 2). The distribution of studies among countries was as follows: Finland 12, Norway 10, Denmark 9, and Sweden 6. We did not identify any studies from Iceland.

**Fig. 3:**
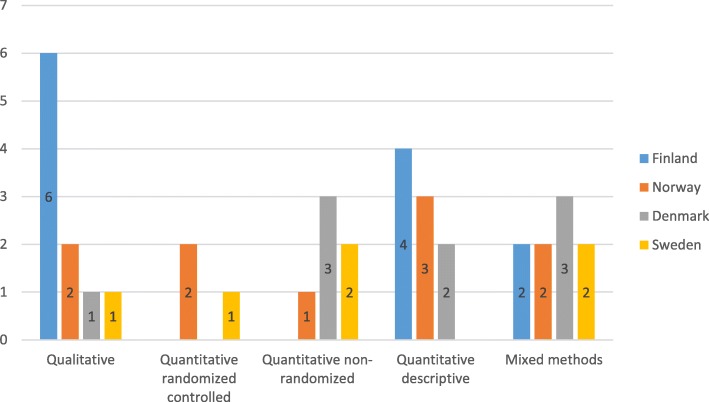
Research design distributed among the Nordic countries (*n* = 37)

An assessment of the research design revealed that most studies used a qualitative or a descriptive design and that a variety of qualitative, quantitative, and mixed methods had been employed (Fig. 3). Qualitative methods included case studies, action research, essays, and focus group interviews, while quantitative methods included surveys and questionnaires, randomized/non-randomized control trials, pre-test and post-test measurements, and observations. Finland has published the greatest number of qualitative studies, and only Sweden and Norway have published randomized control trials.

The participants in the 37 included studies were primarily healthcare professionals and students. The majority of the studies (*n* = 15) included interprofessional teams with participants from several professions. The remaining studies had participants who were either paramedics or nurses/nursing students (*n* = 11), physicians/medical students (*n* = 10), or personnel from pharmacies and health food stores (*n* = 1). The most commonly reported simulation modality [34] was manikins (*n* = 18), followed by simulated patients (*n* = 12), virtual reality (*n* = 4), and computer-based simulators, i.e., learner interaction with only a computer screen-based activity (*n* = 4). Three studies used two simulation modalities [35–37], two studies did not identify the type of simulation modality used [38, 39], and one study compared two simulation modalities [36].

### Themes

The thematic analysis revealed five themes: technical skills, non-technical skills, user experience, educational aspects, and patient safety (Table 3).

**Table 3:** Emerged categories and themes of Nordic simulation studies

Technical skills (*n =* 9)	
Categories	Studies
Resuscitation knowledge and skills	Bjørshol et al. 2011 [42]
Resuscitation knowledge and skills	Creutzfeldt et al. 2012 [41]
Pharmacist and health food clerk technical skills	Hakoinen et al. 2014 [48]
Knowledge and skills in nursing management	Jansson et al. 2014 [46]
Dose and methods of teaching resuscitation	Jäntti et al. 2009 [44]
Resuscitation performance and assessment of simulation method	Mondrup et al. 2011 [40]
Resuscitation knowledge and skills	Naess et al. 2011 [43]
Assessment of learning outcomes and learners’ feedback	Silvennoinen et al. 2016 [45]
Self-assessment of skills in emergency care	Utsi et al. 2008 [47]
Non-technical skills (*n* = 9)	
Categories	Studies
Communication skills assessment	Aspegren et al. 2006 [49]
Interprofessional communication and collaboration	Dahl Pedersen et al. 2006 [56]
Self-assessment of communication skills	Gabrielsen et al. 2016 [50]
Leadership and team skills in cardiac arrest	Høyer et al. 2009 [51]
Leadership skills in interprofessional teams	Jacobsson et al. 2012 [53]
Communication and quality management skills related to patients with chronic diseases	Jensen et al. 2013 [54]
Assessment tool for decision-making	Lauri 1992 [52]
Self-assessment on trauma team management know-how	Rosqvist & Lauritsalo 2013 [55]
Multiprofessional team training	Østergaard et al. 2008 [35]
User experience (*n =* 9)	
Categories	Studies
Participants’ perception of the simulation	Ameur et al. 2003 [63]
Participants’ experience of learning outcome from simulation	Bondevik et al. 2006 [57]
Participants’ experience on physical, psychosocial and organizational factors that affect the CSL learning environment	Haraldseid et al. 2015 [37]
Participants’ experience and self-assessment of three simulation modalities	Koponen & Pyörälä 2014 [62]
Participants’ experience with computer-based virtual simulation	Mäkitie et al. 2008 [64]
Participants’ experience on virtual patients	Salminen et al. 2014 [58]
Participants’ experience on resuscitation course	Thesen et al. 2004 [59]
Participants’ experience on teaching and learning on in-service training	Toivanen et al. 2012 [61]
Participants’ experience and self-assessment	Wisborg et al. 2009 [36]
Educational aspects (*n* = 7)	
Categories	Studies
Planning, preparing and conducting clinical simulations	Jensen et al. 2015 [69]
Simulation as a tool for enhancing reflection	Lestander et al. 2016 [67]
Pilot testing of a virtual reality simulation	Mjelstad et al. 2007 [70]
Assessing simulation teaching methods	Poikela et al. 2015 [66]
Issues and challenges with simulation implementation	Reierson et al. 2015 [38]
Curriculum development on interpersonal communication competences	Saaranen et al. 2015 [68]
Satisfaction with interprofessional team training	Westfelt et al. 2010 [71]
Patient safety (*n =* 3)	
Categories	Studies
Effects of intervention on mortality rate	Fuhrmann et al. 2009 [73]
Prevention of medication and systemic errors	Dieckmann et al. 2014 [72]
Development of a pedagogical design of patient safety curriculum	Tella et al. 2015 [39]

#### Technical skills

This theme of technical skills included nine studies aiming to use simulation to learn technical skills, which is defined as “a skill that is required for the accomplishment of a specific task. In healthcare, it is about the knowledge, skills, and ability to accomplish a specific medical task; for example, inserting a chest tube or performing a physical examination” ([34] p. 39). The results in two of the studies demonstrated that participants improved their knowledge and skills in resuscitation (CPR) by using a manikin [40] or a virtual world multiplayer [41]. However, in a third study, participants exposed to stress performed resuscitation on a manikin with similar skill level as participants who were not exposed to stress [42]. When the quality of advanced CPR training using a manikin was compared to national guidelines, it was found to be satisfactory [43]. Another study evaluated the methods used to teach CPR in institutions that provided instructions at different levels to emergency medical providers. The results demonstrated that the hours of theoretical lessons or training on a manikin varied widely among institutions. In one third of the institutions, the instructor’s visual estimation was the sole method used to teach chest compression rate and depth, while different technical methods were seldom used [44].

In one study, residents’ skills were evaluated using a computer-based simulator (endoscopy simulator) [45]. The findings indicated that the simulation modality facilitated learning of endoscopic skills without morbidity and operating room inefficiency. The transfer of learned skills to clinical practice following SBL using a manikin was observed in the nursing management of patients requiring mechanical ventilation, but the scores of participants’ factual knowledge, evaluated through a multiple-choice questionnaire, did not change statistically [46]. These results may suggest the separation of theory and practice; that is, simulation provides support to the acquisition of motor skills but not to cognitive factual knowledge, if these are learned separately. The evaluation of using a manikin in emergency situations as a method for interprofessional training in primary care found a significant increase in the participants’ confidence in their own roles and in the order of necessary actions [47]. A study that used simulated customers who were purchasing a nutritional supplement to compare patient counseling performance in pharmacies and health food stores, demonstrated that the health food stores provided faster service compared to the pharmacies, a finding that is not surprising given the competing invested interest in the customers. However, information provided to customers in pharmacies was based on scientific facts, while well-being was the primary focus in health food stores [48].

#### Non-technical skills

Nine studies reported using SBL for training in non-technical skills, defined as communication (patient-provider, team), leadership, teamwork, situational awareness, decision-making, resource management, safe practice, adverse event minimization/mitigation, and professionalism [34]. Four studies focused on uniprofessional team training in non-technical skills using SBL [49–52]. In medical education, simulation training resulted in improved communication skills in interviewing the simulated patient [49]. More than half of the nurses in a postoperative care study reported that a simulation-based communication course using a simulated patient improved their comprehension regarding how to use the communication model, and one third reported that their communication skills improved following completion of the course [50]. One study [52] found some inconsistencies in public health nurses’ decision-making process using computer-based simulation with respect to the needs of the child and family; decisions were related more to the developmental stage of the child than to the unique needs of each family. A study that sought to describe physician behavior when serving as team leader in a simulated cardiac arrest during inter-hospital transfer, using a manikin, revealed deficiencies in junior physicians’ skills as team leaders, especially concerning the delegation of tasks to other personnel [51]. Five studies focused on interprofessional team training in non-technical skills using SBL [35, 53–56] by using either a manikin [53, 55], a simulated patient [54], or both simulation modalities [35]. An evaluation of interprofessional education using a simulated patient in the areas of communication and collaboration revealed that nursing and medical students evaluated the education received very positively. The content and structure met the students’ need for interprofessional education pertaining to ward rounds [56]. The results demonstrated that leaders used different techniques to convey their knowledge to the team in order to create a common goal relevant to work priorities. Changes in repertoires were dependent upon the situation and the interaction between team members [53]. Furthermore, Rosqvist and Lauritalo [55] found that most nurses and doctors evaluated the simulation training as useful, irrespective of occupational group, length of work experience, or number of simulation training sessions. The training helped them to remember relevant issues when caring for real trauma patients. In Jensen et al. [54], nurses and physicians benefited from a “Planning and Coordination Module” involved in the planning and coordination of patients with chronic diseases. Østergaard et al. [35] described a framework for the development of a multiprofessional team training course and its connection to patient safety. The use of the framework was illustrated by multiprofessional team training in advanced cardiac life support, trauma team training, and neonatal resuscitation.

#### User experience

The theme of user experience included nine studies describing participants’ evaluation of and experience with simulation and the clinical skills laboratory as a learning environment. One study reported that almost all the medical students viewed the use of a simulated patient as beneficial, valid, realistic, and close to reality, but pointed out the danger of exaggeration and the potential to miss nuances [57]. Another study reported that the medical students regarded virtual simulation as an authentic intermediate learning activity with immediate feedback, a complement to the theoretical and clinical aspects of education, filling in the gaps in clinical knowledge and supporting their self-directed learning and reflective ability [58]. Interprofessional healthcare providers evaluated both theory and practical exercises in a CPR course using a manikin as useful forms of education in their daily work and expressed the desire for repetition of the course once or twice a year [59], which is in line with the European Resuscitation Council Guidelines for Resuscitation [60]. Psychiatric nurses evaluated the simulation with a manikin as a versatile and effective method of training in somatic emergencies and emphasized the teacher’s important role in promoting a positive learning atmosphere, also noting that the nurses’ work profiles were taken into account when designing the simulation scenarios [61]. Two studies evaluated user experience by comparing simulated patients with role-play and theater in education [62], or by comparing simulated patients and simple resuscitation manikins [36]. The studies reported that the participants assessed educational outcome to be high [36, 62], also indicating that it was unrelated to the order and appearance of the simulation modality [36, 62]. The participants in Wisborg et al. [36] felt that the choice between simulation modalities, i.e., manikin or simulated patient, should be determined by the simulated case. In Koponen and Pyörälä [62], the medical students’ self-reported learning outcomes were communication skills, knowledge of doctor-patient communication, patient-centeredness, and enhanced awareness of interpersonal communication competence unrelated to the educational method. Another study reported that a group of medical students exposed to context manipulation (e.g., talking to the manikin as a real patient, reading the description of the patient, wearing operating clothes), perceived the simulation by using a bronchoscopy simulator as more realistic than another group did that focused only on skills training [63]. In Mäkitie et al. [64], four different computer-based virtual models were developed and tested on medical residents, who found the models suitable for learning rough anatomic structures. However, the lack of microscopic details and the rough structures decreased the usefulness of the model. Haraldseid et al. [37] reported that those factors affecting the clinical skills laboratory learning environment included students’ experience of the physical (including manikins and simulated patients), psychosocial, and organizational factors.

#### Educational aspects

The seven studies in this theme were about designing SBL activities involving preparation (including choice of simulation modalities), briefing, simulation activity, debriefing or feedback, reflection, and evaluation [65]. In one quasi-experimental study related to nursing education, the findings revealed that nursing students who used a computer-based simulation program were more likely to report meaningful learning themes than those who first were exposed to the lecture method [66]. Lestander et al. [67] explored the value of reflection after simulation using a manikin when a three-step post-simulation reflection model was used (1. individual written reflection, 2. verbal group reflection, and 3. individual written reflection). The main theme in the first reflection was “Starting to act as a nurse” and in the second “Maturing in the profession” indicating that repeated reflections stimulate and enhance student learning. Development and implementation of the “Practical Skill Performance model” in nursing education revealed six issues and challenges: anchoring the research in the faculty structure, repeated dialog meetings between research group and faculty, adapting teacher and student roles in the clinical skills laboratory, unequivocal understanding of the model as a theoretical and normative learning tool, curriculum consistency, and teachers’ engagement and enthusiasm [38]. Simulation modality was not stated. In a study related to master’s education coursework in health sciences, Saaranen et al. [68] found that planning of teaching, carrying out different stages of the simulation exercise by using simulated patients, participant roles, and students’ personal factors were central to the attainment by the students of competence in communication.

Three studies focused on the development of interprofessional simulation. The first study described a methodological approach to the planning, preparing, and conducting of clinical simulations for doctors and nurses by using simulated patents. Unintended consequences concerning terminology and changes in the division of responsibility among healthcare professionals were identified and questions were raised concerning future workflow across sector borders [69]. The second study described a pilot study that employed virtual reality simulation to train trauma teams in leadership, communication, and coordination. When comparing students with specialists, it was not surprising that the students executed fatal interventions during treatment of a multi-trauma patient, while the specialist groups were faster and stabilized the patient without ordering a CT before life-threatening bleeding was clarified [70]. The third study presented healthcare professionals’ perceptions of collaboration and safety in the emergency department and demonstrated how interprofessional teamwork could be implemented to improve safety [71]. Although the participants evaluated as very satisfactory the simulation training using a manikin, the nurses and nurse assistants addressed the need for deeper collaboration by employing an established training method in their own working environment.

#### Patient safety

The patient safety theme was the focus of three studies about prevention of medication and systemic errors [72], the effects of an intervention on mortality rates [73], and user experience in the pedagogical design of a patient safety curriculum [39]. A manikin was used in two of the studies [72, 73], while in the third study, the simulation modality was not stated [39]. Dieckmann et al. [72] found that the design of labels benefited from the standardized construction of the labels, while the complexity of the system and different meanings of colors posed challenges when considered in conjunction with the actual application context. Fuhrmann et al. [73] found no effect of multi-professional SBL, focusing on recognition and management of the deteriorating patient, on incidence of patients with abnormal vital signs, staff awareness of patients at risk, 30-day mortality, and length of hospital stay among patients at risk. Finally, Tella et al. [39] found that nursing students considered patient safety education that used simulation to be more valuable for their own learning than educational materials provided by their programs.

## Discussion

The primary strength of SBL research in the Nordic literature is that the studies cover a wide range of themes, such as technical skills, non-technical skills, user experience, educational aspects, and patient safety. This demonstrates a broad interest in the field. In addition, most studies include interprofessional teams made up of healthcare professionals and students. We identified a number of simulation studies from all Nordic countries except Iceland. Among the Nordic countries, Finland stood out with the largest number of studies, especially those that used a variety of simulation modalities—for example, simulated patients [62], manikins [55], computer-based simulator [52], and virtual reality simulator [45]. Overall, an assessment of research designs revealed that qualitative and quantitative descriptive studies were employed most frequently.

The results from the nine studies categorized under the *Technical skills* theme demonstrated that skills can be improved by computer-based simulations. In these studies, the process of learning technical skills through simulation was researched in various contexts to measure and evaluate skills, educational methods, and safety as well as quality of service. However, only one of the studies showed a transfer of skills from simulation to clinical practice. These results are similar to the findings in other international review studies [3, 74–76], which reported improvement after SBL training in procedure and task performance, patient comfort and complication rates.

Regarding the theme *Non-technical skills*, nine studies demonstrated that interprofessional as well as uniprofessional training improved communication and collaboration skills when using SBL. The studies also revealed that leadership and communication styles depend on the context and participants’ competence. This finding is congruent with the results of a previous non-Nordic study which found a number of factors that had an impact on the output of health professional teamwork training, i.e., the context in which the program was delivered, starting points of individual learners, and the opportunity to transfer new learning into practice [11]. A recent international review [74] also demonstrated positive patient outcomes after team and non-technical skills training, such as quicker intubation with fewer complications [77] and lower mortality [78]. However, none of the Nordic studies included in this review have reached such promising conclusions.

The nine studies categorized under the *User experience* theme demonstrated participant satisfaction with SBL and with the educational methods used, which were reputed to be valid and realistic. Results from studies of experiences by nursing students partially support the findings of our review, i.e., students were satisfied with SBL [9]. Unlike the results of our review, however, nursing students’ experience in Foronda ([9], pp. e412) demonstrated that SBL also caused anxiety or stress, which might influence learning.

Under the theme *Educational aspects*, the seven studies revealed lessons learned when designing SBL. The results demonstrated promotion of learning and reflections that are meaningful in SBL compared to traditional lectures, challenges and possibilities for use in clinical practice, and simulation through the implementation of new models in skill training and the design of interprofessional team training. Additionally, the educational outcome was not dependent on the simulation modality, but rather, on the simulated case and the ability of the teacher to create a safe learning environment. The educational aspects of SBL, for example, design of SBL in the curriculum including reflections, and solving challenges with implementing SBL, are also emphasized as critical conditions for learning in the international literature [79, 80]. An earlier international review has indeed shown that a strong instructional format—that is, how a course is designed as well as feedback from the instructor—results in higher learning outcomes [81].

Only three studies were categorized under the theme *Patient safety*. Unfortunately, the intervention in Fuhrmann et al. [73] showed no improvement in terms of patient survival or staff awareness of patients at risk on general wards. Incorrect design of medication labels might put patient safety at risk, and additional patient safety education in nursing education would be valuable in preparing for clinical practice. Previous international research has supported the value of healthcare simulation in enhancing patient safety [82]. The expert group in Sollid et al. [82] identified the following five topics in healthcare simulation that contribute the most to improving patient safety: technical skills, non-technical skills, assessment, effectiveness, and system probing. Evidence of positive patient outcomes is, however, emerging in the literature, and non-Nordic systematic reviews have demonstrated small positive effects on patient-related outcomes and positive effects on learning and skills transfer to the clinical environment [74].

This review revealed that most of the Nordic simulation literature lacks robust research evidence that supports simulation as an effective educational method. RCT design was used only in Norway [36, 42] and Sweden [46], which implies that future simulation studies in the Nordic region may benefit from strengthening the research design and methodology. Furthermore, most of the studies that used simulation in learning technical skills included a small sample size and employed a descriptive design. Similar methodological and design shortcomings have been identified in the recent international literature [74, 75, 81]. Consequently, SBL research that addresses the lack of robust research design and methodology should continue to point in the right direction. Interprofessional team training using SBL for non-technical skills has become increasingly important because of the changing healthcare system, both in and out of the hospital as well as in primary care [83]. Patient care becomes a series of transitions from home to hospital to rehabilitation facilities and back to home again, necessarily engaging a host of multidisciplinary professionals—nurses, doctors, etc., who must work together to provide a seamless web of health services. Five of the nine studies in the *Non-technical skills* theme were performed in Denmark, which suggest a need for further research in this theme in the other Nordic countries. Another gap identified is the lack of Danish studies in the *User experience* theme; only one was identified (Table 2). Furthermore, limited research that addressed patient safety was found. Fuhrmann et al.’s [73] considerable work linking the effects of simulation to patient outcomes may provide direction for future targeted interventions to decrease adverse events and patient mortality. However, evidence of patient outcomes linked to SBL is very difficult to demonstrate because of many other external medical factors that can influence patient outcomes and that are unrelated to the effects of SBL [84]. Therefore, the following question is anticipated: Is it really necessary to demonstrate patient outcomes to support the use of SBL in healthcare?

### Future directions

No study identified a cross-country comparison of studies using simulation in the Nordic countries. This stands out as an important area for future research and corresponds well with the NordForsk strategy for improved research collaboration [25]. The goal of the NordForsk strategy is to enhance the quality, impact, and cost-efficiency of Nordic research and research infrastructure collaboration, for example, by strengthening integrated cross-sectoral research and supporting the establishment of new joint Nordic research infrastructures. Another gap identified is the scarce number of studies from the primary care setting or from a combination of the specialized and primary care settings. The lack of simulation studies that use a primary care perspective mirrors findings from the international literature [85], adding emphasis to the importance of knowledge development in this area. According to our review, no research exists that focuses on valid assessment methods, scales, and rating instruments for non-technical skills, all of which would enable cross-country research collaboration in the Nordic countries. There is also a lack of simulation research that studies cost-effectiveness in the Nordic countries. The demonstration of cost-effectiveness could increase the resources directed to SBL. In this review, 11 studies stated funding from different private and public funds. Another gap identified in the Nordic literature is the lack of research on in situ trauma team simulation training in the hospital setting. This type of research would be beneficial because it is conducted in the actual patient care environment, using equipment and resources from that unit, and involving actual members of the healthcare team [86]. In situ training makes the learning context authentic, possibly reinforcing the learning experience.

### Limitations

The review has several limitations. Our search strategy did not identify all critical articles. Only five international, one Nordic, and one Finnish databases were searched. Other databases may have revealed additional articles. The review focused exclusively on peer-reviewed simulation studies in healthcare education in the Nordic countries. Due to inclusion criteria, gray literature from the Nordic countries was not included. However, we believe that this integrative review provides new insights and offers possible directions for future research. A promising strength of this review is the close collaboration among the authors, who represented the Nordic countries in all phases of the review process, especially in the thematic analysis.

## Conclusion

Most Nordic research literature on SBL has employed a qualitative or a descriptive design that includes interprofessional or uniprofessional teams and relates to technical skills, non-technical skills, user experience, educational aspects, and patient safety. Shortcomings in simulation research in the Nordic countries include a lack of randomized control trials and evidence that supports simulation as an educational method, as well as a dearth of studies focusing on patient safety, the primary care setting, or a combination of specialized and primary care settings. Suggested directions for future research include strengthening the design and methodology of SBL studies, incorporating a cross-country comparison of studies using simulation in the Nordic countries, and studies combining specialized and primary care settings.

## Additional files

Additional file 1: Search in databases, terms, and hits. (DOCX 18 kb)

Additional file 2: Mixed methods appraisal tool [32] used for the quality appraisal (*N* = 37). (DOCX 48 kb)
