# Supplementary material for: Status of Nordic research on simulation-based learning in healthcare: an integrative review
Source: Adv Simul (Lond). 2018 Jul 4;3:12. doi: 10.1186/s41077-018-0071-8 (PMC6032768; doi:10.1186/s41077-018-0071-8)
Supplement: Supplementary file 1 — Search in databases, terms, and hits. (DOCX 18 kb) [file 41077_2018_71_MOESM1_ESM.docx]

Additional file 1. Search in databases, terms and hits

| **Initial search (June 2016)** | | |
| --- | --- | --- |
| Terms (limited to abstract) | Databases | Hits |
| S1: “Nordic” or “Norway” or “Sweden” or “Finland” or “Denmark” or “Iceland” | Academic Search Premier (ASP)  CINAHL  ERIC  Medline  SocINDEX | 107,285  11,853  0  80,639  16,182 |
| S2: “Health care” or “nursing” or “medicine” | ASP  CINAHL  ERIC  Medline  SocINDEX | 462,882  201,939  0  519,297  57,796 |
| S3: “simulation” or “teaching” or “learning” or “curriculum” or “assessment” or “examination” | ASP  CINAHL  ERIC  Medline  SocINDEX | 1,374,439  184,402  519,891  1,350,994  0 |
| Combined search S1+S2+S3 | ASP  CINAHL  ERIC  Medline  SocINDEX | 627  478  56  1,183  0 |
|  | Duplicates removed  Total number of hits screened | 1,493  1,378  1,360 excluded  18 included |
| **Second search** | | |
| Simulation training (MeSH) | Svemed+ (Scandinavian database) | 48 hits  32 excluded  (17 not peer reviewed,  1 Correspondence,  4 Commentary,  6 Letter or Debate,  2 Education,  2 Proceedings, and  3 reviews)  13 included |
| Simulation  Simulation training | Medic (Finnish database) | 102 hits  3 duplicates removed  87 excluded  (30 not peer reviewed, 23 dissertations,  10 master's thesis,  1 book,  1 comment,  2 editorials,  1 duplicate,  22 irrelevant, and  3 reviews)  6 included |
|  |  | **A total of 37 articles included** |
