# Supplementary material for: Status of Nordic research on simulation-based learning in healthcare: an integrative review
Source: Adv Simul (Lond). 2018 Jul 4;3:12. doi: 10.1186/s41077-018-0071-8 (PMC6032768; doi:10.1186/s41077-018-0071-8)
Supplement: Supplementary file 2 — Mixed methods appraisal tool [32] used for the quality appraisal (N = 37). (DOCX 48 kb) [file 41077_2018_71_MOESM2_ESM.docx]

Additional file 2: Mixed methods Appraisal Tool [32] used for the quality appraisal (N=37)

The studies included in the review are organized alphabetical in relation to study designs (1. Qualitative, 2.Quantitative randomized controlled trials, 3. Quantitative non-randomized, 4. Quantitative descriptive, and 5. Mixed methods).

Comments are provided in studies written in Finnish language

| Question | Dieckmann et al. 2016 | | Hakoinen et al. 2014 | | Haraldseid et al. 2015 | | Lestander et al. 2016 | | Mäkitie et al. 2008 | | Poikela et al. 2015 | | Reierson et al. 2013 | | Saaranen et al. 2015 | |
| --- | --- | --- | --- | --- | --- | --- | --- | --- | --- | --- | --- | --- | --- | --- | --- | --- |
|  | Y/N | Comments | Y/N | Comments | Y/N | Comments | Y/N | Comments | Y/N | Comments | Y/N | Comments | Y/N | Comments |  | Comments |
| Screening questions  (for all types) |  |  |  |  |  |  |  |  |  |  |  |  |  |  |  |  |
| Are there clear qual. and quant. research questions (RQ) (or objectives), or a clear mixed methods question (or objective)? | Y |  | Y |  | Y |  | Y |  | N | Only the aim is described. | Y |  | Y |  | Y |  |
| Do the collected data allow address the RQ (objective)? E.g., consider whether the follow-up period is long enough for the outcome to occur. | Y |  | Y |  | Y |  | Y |  | Y | Data collection is described. | Y |  | Y |  | Y |  |
| **1.Qualitative**  1.1 Are the sources of qual. data relevant to address the RQ? | Y |  | Y | Data set is small. | Y |  | Y |  | Y | Process of rapid prototyping and manufacturing the three models tested is well described. | Y |  | Y |  | Y |  |
| 1.2 Is the process for analyzing qual. data relevant to address the RQ? | Y |  | Y |  | Y |  | Y |  | Y | RQs not mentioned explicit | Y |  | Y |  | Y |  |
| 1.3 Is appropriate consideration given to how findings relate to the context, e.g. the setting, in which the data were collected? | Y |  | Y | Well described | Y |  | Y |  | Y | Authors describe the experiences of models and suggest how to make improvements in the future. | Y |  | Y |  | Y |  |
| 1.4 Is appropriate consideration given to how findings relate to researchers’ influence, e.g. through their interactions with participants? | N | Did not find considerations on researchers influence | N | Described very briefly | Y |  | N |  | Y | Improvement ideas are suggested and future challenges described. | Y | Limitations addressed | Y | Limitations addressed | N |  |
| Scoring | 75% | | 75% | | 100% | | 75% | | 100% | | 100% | | 100% | | 75% | |
| Question | Salminen et al. 2014 | | Toivanen et al. 2012 | |  | |  | |  | |  | |  | |  | |
|  | Y/N | Comments | Y/N | Comments |  |  |  |  |  |  |  |  |  |  |  |  |
| Screening questions (for all types) |  |  |  |  |  |  |  |  |  |  |  |  |  |  |  |  |
| Are there clear qual. and quant. research questions (RQ) (or objectives), or a clear mixed methods question (or objective)? | Y |  | Y | Three specific research questions are described. |  |  |  |  |  |  |  |  |  |  |  |  |
| Do the collected data allow address the RQ (objective)? E.g., consider whether the follow-up period is long enough for the outcome to occur. | Y |  | Y | Group interviews were used. The data collection is described properly. |  |  |  |  |  |  |  |  |  |  |  |  |
| **1.Qualitative**  1.1 Are the sources of qual. Data relevant to address the RQ? | Y |  | Y | All participants had participated in the somatic emergency patient simulation education. |  |  |  |  |  |  |  |  |  |  |  |  |
| 1.2 Is the process for analyzing qual. Data relevant to address the RQ? | Y |  | Y | The analysis process is described in the figure. |  |  |  |  |  |  |  |  |  |  |  |  |
| 1.3 Is appropriate consideration given to how findings relate to the context, e.g. the setting, in which the data were collected? | Y |  | N | Those who had positive experiences might be the same persons who volunteered in the study. The time between the education and interviews were 5 months, which may affect the findings. |  |  |  |  |  |  |  |  |  |  |  |  |
| 1.4 Is appropriate consideration given to how findings relate to researchers’ influence, e.g. through their interactions with participants? | Y |  | Y | The researches’ possible influence through their interaction with participants in group interview situations is not mention. |  |  |  |  |  |  |  |  |  |  |  |  |
| Scoring | 100% | | 100% | |  |  |  |  |  |  |  |  |  |  |  |  |

| Question | Bjørshol et al. 2011 | | Jansson et al. 2014 | | Wisborg et al. 2009 | |
| --- | --- | --- | --- | --- | --- | --- |
|  | Y/N | Comments | Y/N | Comments | Y/N | Comments |
| Screening questions (for all types) |  |  |  |  |  |  |
| Are there clear qual. and quant. research questions (RQ) (or objectives), or a clear mixed methods question (or objective)? | Y |  | Y |  | Y |  |
| Do the collected data allow address the RQ (objective)? E.g., consider whether the follow-up period is long enough for the outcome to occur. | Y |  | Y |  | Y |  |
| **2. Quantitative randomized controlled (trials)**  2.1 Is there a clear description of the randomization? | Y |  | Y |  | Y |  |
| 2.2 Is there a clear description of the allocation concealment? | Y | No blinding was possible,  unaware of the differences  between the two conditions. | Y |  | Y |  |
| 2.3 Are there complete outcome data? | Y |  | Y |  | Y |  |
| 2.4 Is there low withdrawal/drop-out? | Y |  | N | Small sample size and high drop-out | Y |  |
| Scoring | 100% | | 75% | | 100% | |
|  |  | |  | |  | |

| Question | Aspegren et al. 2006 | | Creutzfeldt et al. 2012 | | Fuhrmann et al. 2009 | | Mjelstad et al. 2007 | | Westfelt et al. 2010 | | Østergaard et al. 2008 | |  | |  | |
| --- | --- | --- | --- | --- | --- | --- | --- | --- | --- | --- | --- | --- | --- | --- | --- | --- |
|  | Y/N | Comments | Y/N | Comments | Y/N | Comments | Y/N | Comments | Y/N | Comments | Y/N | Comments |  |  |  |  |
| Screening questions (for all types) |  |  |  |  |  |  |  |  |  |  |  |  |  |  |  |  |
| Are there clear qual. and quant. research questions (RQ) (or objectives), or a clear mixed methods question (or objective)? | Y |  | Y | hypotheses | Y |  | Y |  | Y |  | Y |  |  |  |  |  |
| Do the collected data allow address the RQ (objective)? E.g., consider whether the follow-up period is long enough for the outcome to occur. | Y |  | Y |  | Y |  | Y |  | Y |  | Y |  |  |  |  |  |
| **3. Quantitative non-randomized**  3.1 Are participants recruited in a way that minimizes selection bias? | Y |  | Y | volunteered | Y |  | N |  | N |  | N |  |  |  |  |  |
| 3.2 Are measurements appropriate regarding the exposure/intervention and outcomes? | Y |  | Y |  | Y |  | Y |  | Y |  | Y |  |  |  |  |  |
| 3.3 In the groups being compared, are the participants comparable, or do researchers take into account the difference between groups? | Y |  | Y |  | N | Unsure | N |  | Y |  | Not stated |  |  |  |  |  |
| 3.4 Are there complete outcome data, and, when applicable, an acceptable response rate, or an acceptable follow-up rate for cohort studies? | Y |  | Y |  | N | Unsure | Y |  | Y |  | N |  |  |  |  |  |
| Scoring | 100% | | 100% | | 50% | | 50% | | 75% | | 25% | |  | |  | |

| Question | Gabrielsen et al. 2016 | | Høyer et al. 2009 | | Jäntti et al. 2009 | | Lauri 1992 | | Mondrup et al. 2011 | | Naess et al. 2011 | | Silvennoinen et al. 2016 | | Tella et al. 2015 | |
| --- | --- | --- | --- | --- | --- | --- | --- | --- | --- | --- | --- | --- | --- | --- | --- | --- |
|  | Y/N | Comments | Y/N | Comments | Y/N | Comments | Y/N | Comments | Y/N | Comments | Y/N | Comments | Y/N | Comments | Y/N | Comments |
| Screening questions (for all types) |  |  |  |  |  |  |  |  |  |  |  |  |  |  |  |  |
| Are there clear qual. and quant. research questions (RQ) (or objectives), or a clear mixed methods question (or objective)? | Y |  | Y |  | Y |  | Y |  | Y |  | Y |  | N | RQ not stated | Y |  |
| Do the collected data allow address the RQ (objective)? E.g., consider whether the follow-up period is long enough for the outcome to occur. | Y |  | Y |  | Y |  | Y |  | Y |  | Y |  | N | The aim of the endoscopy training is missing | Y |  |
| **4. Quantitative descriptive**  4.1 Is the sampling strategy relevant to address the quant. RQ? | Y |  | N | Sampling not stated | Y |  | Y |  | Y |  | Y |  | Y | Data of six participants were analyzed | Y |  |
| 4.2 Is the sample representative of the population under study? | N |  | N | Not stated | Y |  | Y |  | N |  | N |  | N |  | Y |  |
| 4.3 Are measurements appropriate? | Y |  | Y |  | Y |  | Y |  | Y |  | Y |  | Y |  | Y |  |
| 4.4 Is there an acceptable response rate? | Y |  | N | Not stated | Y |  | Y |  | Y |  | Y |  | N | Only data from six out of 16 participants were analyzed | Y |  |
| Scoring | 75% | | 25% | | 100% | | 100% | | 75% | | 75% | | 50% | | 100% | |
|  | | | | | | | | | | | | | | | | |
| Question | Thesen et al. 2004 | |  | |  | |  | |  | |  | |  | |  | |
|  | Y/N | Comments |  |  |  |  |  |  |  |  |  |  |  |  |  |  |
| Screening questions (for all types) |  |  |  |  |  |  |  |  |  |  |  |  |  |  |  |  |
| Are there clear qual. and quant. research questions (RQ) (or objectives), or a clear mixed methods question (or objective)? | Y |  |  |  |  |  |  |  |  |  |  |  |  |  |  |  |
| Do the collected data allow address the RQ (objective)? E.g., consider whether the follow-up period is long enough for the outcome to occur. | Y |  |  |  |  |  |  |  |  |  |  |  |  |  |  |  |
| **4. Quantitative descriptive**  4.1 Is the sampling strategy relevant to address the quant. RQ? | Y |  |  |  |  |  |  |  |  |  |  |  |  |  |  |  |
| 4.2 Is the sample representative of the population under study? | N |  |  |  |  |  |  |  |  |  |  |  |  |  |  |  |
| 4.3 Are measurements appropriate? | Unsure |  |  |  |  |  |  |  |  |  |  |  |  |  |  |  |
| 4.4 Is there an acceptable response rate? | Y |  |  |  |  |  |  |  |  |  |  |  |  |  |  |  |
| Scoring | 50% | |  |  |  |  |  |  |  |  |  |  |  |  |  |  |

| Question | Ameur et al. 2003 | | Bondevik et al. 2006 | | Dahl Pedersen et al. 2006 | | Jacobsson et al. 2012 | | Jensen et al. 2013 | | Jensen et al. 2015 | | Koponen & Pyörälä 2014 | | Rosqvist & Lauritsalo 2013 | |
| --- | --- | --- | --- | --- | --- | --- | --- | --- | --- | --- | --- | --- | --- | --- | --- | --- |
|  | Y/N | Comments | Y/N | Comments | Y/N | Comments | Y/N | Comments | Y/N | Comments | Y/N | Comments | Y/N | Comments | Y/N | Comments |
| Screening questions (for all types) |  |  |  |  |  |  |  |  |  |  |  |  |  |  |  |  |
| Are there clear qual. and quant. research questions (RQ) (or objectives), or a clear mixed methods question (or objective)? | Y |  | Y |  | Y |  | Y |  | Y |  | Y |  | N | Unclear | Y | RQ mixed |
| Do the collected data allow address the RQ (objective)? E.g., consider whether the follow-up period is long enough for the outcome to occur. | Y |  | Y |  | Y |  | Y |  | Y |  | Y |  | Y |  | Y |  |
| **5. Mixed methods**  5.1 Is the mixed methods research design relevant to address the qual. and quant. RQ? | Y |  | Y |  | Y |  | Y |  | Y |  | Y |  | Y |  | Y |  |
| 5.2 Is the integration of qual. and quant. data relevant to address the RQ? | Y |  | Y |  | Y |  | Y |  | Y |  | Y |  | Y |  | Y |  |
| 5.3 Is appropriate consideration given to the limitations associated with this integration in a triangulation design? | N | Only a small sample | N |  | N |  | Y |  | N |  | Y |  | Y |  | Y |  |
| Scoring | 66% | | 66% | | 66% | | 100% | | 66% | | 100% | | 100% | | 100% | |
|  | | | | | | | | | | | | | | | | |
| Question | Utsi et al. 2008 | |  | |  | |  | |  | |  | |  | |  | |
|  | Y/N | Comments |  |  |  |  |  |  |  |  |  |  |  |  |  |  |
| Screening questions (for all types) |  |  |  |  |  |  |  |  |  |  |  |  |  |  |  |  |
| Are there clear qual. and quant. research questions (RQ) (or objectives), or a clear mixed methods question (or objective)? | Y |  |  |  |  |  |  |  |  |  |  |  |  |  |  |  |
| Do the collected data allow address the RQ (objective)? E.g., consider whether the follow-up period is long enough for the outcome to occur. | Y |  |  |  |  |  |  |  |  |  |  |  |  |  |  |  |
| **5. Mixed methods**  5.1 Is the mixed methods research design relevant to address the qual. and quant. RQ? | Y |  |  |  |  |  |  |  |  |  |  |  |  |  |  |  |
| 5.2 Is the integration of qual. and quant. data relevant to address the RQ? | Y |  |  |  |  |  |  |  |  |  |  |  |  |  |  |  |
| 5.3 Is appropriate consideration given to the limitations associated with this integration in a triangulation design? | N |  |  |  |  |  |  |  |  |  |  |  |  |  |  |  |
| Scoring | 66% | |  |  |  |  |  |  |  |  |  |  |  |  |  |  |
| Yes-Y, No-N | | | | | | | | | | | | | | | | |
